# Increased fecal calprotectin levels in Crohn’s disease correlate with elevated serum Th1- and Th17-associated cytokines

**DOI:** 10.1371/journal.pone.0193202

**Published:** 2018-02-21

**Authors:** Arno R. Bourgonje, Julius Z. H. von Martels, Paul de Vos, Klaas Nico Faber, Gerard Dijkstra

**Affiliations:** 1 Department of Gastroenterology and Hepatology, University of Groningen, University Medical Center Groningen, Groningen, The Netherlands; 2 Department of Pathology and Medical Biology, University of Groningen, University Medical Center Groningen, Groningen, The Netherlands; University Hospital Llandough, UNITED KINGDOM

## Abstract

**Background:**

Patient-reported symptoms and endoscopic disease activity do not correlate well in Crohn’s disease (CD). This warrants the need for reliable biomarkers to early detect active intestinal inflammation. Currently, the fecal calprotectin level is the most commonly used biomarker for inflammatory activity in CD. However, the diagnostic accuracy of the fecal calprotectin level is not fully efficacious and diagnosis may be further improved by the identification of other biomarkers for active CD. Here, we studied the association of a variety of serum disease markers with fecal calprotectin levels in CD patients.

**Methods:**

39 CD patients were included and subdivided into ‘normal’ (defined as < 200 mg/kg feces) and ‘increased’ (defined as > 200 mg/kg feces) fecal calprotectin level groups. Serum levels of 37 different cytokines, chemokines and markers for angiogenesis and vascular injury were quantified by an electrochemiluminescence multiplex assay (V-PLEX Human Biomarker 40-Plex Kit of Meso Scale Discovery ^®^). Correlations between individual biomarkers and the fecal calprotectin level were assessed using Spearman’s correlation coefficient (*ρ*).

**Results:**

A highly significant positive correlation was observed between the pro-inflammatory serum cytokines IFN-γ and CRP and fecal calprotectin levels (*P* < 0.01). Moreover, fecal calprotectin levels showed a significant positive correlation with IL-6, TNF-β, SAA and IL-17A (*P* < 0.05).

**Conclusion:**

We show that a positive correlation exists between multiple serum Th1- and Th17-associated cytokines and the fecal calprotectin level. These cytokines and CRP may serve as additional biomarkers for determining disease activity and evaluating treatment response in CD. Ultimately, this may result in more efficient treatment of active disease in CD patients and prevention of complications.

## Introduction

Crohn’s disease (CD) is a chronic inflammatory disease that mainly affects the gastro-intestinal tract and is characterized by an inappropriate and ongoing immune response. [1] Most patients suffer from a relapse-remitting disease course that is difficult to predict. [2,3] Predicting the disease course is hampered by the poor availability of adequate disease biomarkers or symptoms that forecast a flare of inflammation. Longstanding sub-clinical disease activity increases the risk of various severe complications, such as stricturing or penetrating disease (i.e. fistula or abscess formation). [4] Appropriate and prompt treatment of the inflammatory activity lowers the risk of these severe complications, and thus prevents future surgical interventions.

Currently, the only reliable approach for diagnosis of CD is an invasive ileo-colonoscopy. However, this procedure has several disadvantages, such as the risk of perforation, bleeding, relatively high costs and, most importantly, a high patient burden. Furthermore, a poor association exists between patient-reported symptoms and the observed endoscopic inflammatory activity. For instance, clinical risk scores, such as the Harvey Bradshaw Index (HBI) or the Crohn’s Disease Activity Index (CDAI), cannot accurately predict active intestinal inflammation. [5,6]

In clinical practice, fecal calprotectin levels are commonly used as non-invasive biomarker that significantly correlates with inflammatory disease activity and response to therapy. [5,7–10] Calprotectin is a 36 kDa calcium- and zinc-binding protein dimer (consisting of S100A8 and S100A9) complex present in the cytosol of neutrophilic granulocytes. [11,12] Fecal calprotectin levels are representative of neutrophil migration into the intestinal mucosa that occurs in the process of intestinal inflammation. Despite its proven association with disease activity, the diagnostic accuracy may be further improved by inclusion of additional biomarkers for active inflammation and/or tissue injury in CD. [12–14] Many cytokines have been shown to be involved in disease pathogenesis and might give a more accurate representation of the inflammatory activity in CD relapses in combination with fecal calprotectin levels. Ultimately, this may aid in an improved detection of active disease and a more versatile and effective treatment.

A Th1-driven immune response with increased Th1-associated cytokines such as IFN-γ and TNF-α plays a pivotal role in the pathogenesis of CD. [15] The importance of Th1-responses is also reflected by the clinical use of TNF-α antagonists, such as infliximab, adalimumab and certolizumab, which are effective treatment modalities in CD. [16] Previously, quantification of cytokines for diagnosis of disease has been cumbersome due to low serum concentrations of the relevant cytokines, but new techniques enable us to quantify most cytokines in a highly sensitive, reproducible and validated manner. [17–19]

The aim of this study is to identify potential serum cytokines, chemokines and markers for angiogenesis and vascular injury that might serve as additional biomarkers for inflammatory disease activity in CD. A positive correlation between specific serum biomarkers and fecal calprotectin levels might enhance the diagnostic potential for early recognition of disease exacerbations.

## Methods

### Study population

Patients aged 19–67 years with an established diagnosis of Crohn’s disease (CD) were included from March 2016 to April 2017 at the University Medical Center Groningen (UMCG), the Netherlands. In total, 39 CD patients were included and divided into two groups according to inflammatory disease activity, as determined by fecal calprotectin levels. Patients having a calprotectin level below 200 mg/kg were defined as the ‘normal’ calprotectin group (indicative of remissive disease) and patients with calprotectin levels above 200 mg/kg were defined as the ‘increased’ calprotectin group (indicative of inflammatory active CD). Clinically relevant data were obtained from medical records: age, gender, BMI, smoking history, maintenance medication, Montreal score, ileocecal resection and laboratory parameters. Fecal calprotectin levels were determined in the laboratory of the UMCG as a routine measurement. Serum samples were obtained after patients gave written informed consent (study approved by the Medical Ethical Committee of the UMCG).

### Measurement of cytokines, chemokines and markers for angiogenesis and vascular injury

Serum samples from all patients were collected and stored in 1 mL aliquots in the freezer (-20°C). After thawing, serum samples were centrifuged for 3 minutes at 2000 *g* to remove particulates prior to sample preparation and analysis. Measurement of serum levels of cytokines, chemokines and markers for angiogenesis and vascular injury was performed by the electrochemiluminescence (ECL) multiplex assay (Meso Scale Discovery (MSD ^®^), Meso Scale Diagnostics, Rockville, MD). The MSD V-plex Pro-inflammatory panel 1 (IFN-γ, IL-1β, IL-2, IL-4, IL-6, IL-8, IL-10, IL-12p70, IL-13 and TNF-α), Cytokine panel 1 (GM-CSF, IL-5, IL-7, IL-12/23p40, IL-15, IL-16, IL-17A and TNF-β), Chemokine panel 1 (Eotaxin, MIP-1β, Eotaxin-3, TARC, IP-10, MIP-1α, MCP-1 and MDC), Angiogenesis panel 1 (VEGF, VEGF-C, VEGF-D, Tie-2, Flt-1, PIGF and bFGF) and Vascular injury panel 1 (SAA, CRP, VCAM-1 and ICAM-1) were used to detect a total of 37 molecules. Calibration curves were created in order to calculate serum biomarker concentrations. Calibrator signals were fitted to a 4-parameter logistic model with 1y2 weighting, providing the assay with a wide dynamic range of detection. Biomarker concentrations were calculated by back-fitting ECL signals to the calibration curves. Determination of final concentrations was performed using the MSD Discovery Workbench ^®^ analysis software. Concentrations of all molecules were above the lower limit of detection (LLOD). For the V-plex Pro-inflammatory panel 1, Cytokine panel 1 and Angiogenesis panel 1 assays, standard volumes of 50 μL of each serum sample were added and diluted 2-fold. For the Chemokine panel 1, samples were diluted 4-fold. For the Vascular injury panel 1, a standard volume of 25 μL of each serum sample was added and 1000-fold diluted.

### Statistics

Study population characteristics were presented as means ± standard errors (SEM) or proportions (%, *n*). Serum biomarker distributions were presented as median ± interquartile ranges (IQR) and shown in boxplots (10^th^-90^th^ percentiles) grouped by inflammatory disease activity, as determined by the fecal calprotectin level. Correlations between fecal calprotectin levels and serum biomarker levels were established using the nonparametric Spearman’s correlation coefficient (*ρ*). Data were analyzed using SPSS Statistics 23.0 software package for Windows. *P*-values ≤ 0.05 were considered as statistically significant.

## Results

Characteristics of the study population are presented in Table 1. Patients with a ‘normal’ fecal calprotectin level (*n* = 22) had a mean age of 40.5 ± 2.4 years and consisted of 5 males (22.7%) and 17 females (77.3%). Patients with an increased level of fecal calprotectin (*n* = 17) had a mean age of 39.7 ± 3.4 years and consisted of 5 males (29.4%) and 12 females (70.6%). Patients with increased fecal calprotectin levels had significantly higher C-reactive protein (CRP) levels (*P* < 0.01) and higher erythrocyte sedimentation rates (ESR) (*P* < 0.05) as compared to patients with fecal calprotectin levels in the normal range. No significant differences between groups were observed for disease location, medication use, smoking habits, surgery history and standard laboratory parameters.

**Table 1:** Study population characteristics (*n* = 39) of Crohn’s disease patients with ‘normal’ (< 200 mg/kg) and increased (> 200 mg/kg) fecal calprotectin levels.

Characteristics	Calprotectin < 200 mg/kg (*n* = 22)	Calprotectin > 200 mg/kg (*n* = 17)	*P* value
**Age (years)**	40.5 ± 2.4	39.7 ± 3.4	0.788
**Male gender**	5 (22.7)	5 (29.4)	0.721
**BMI (kg/m^2^)**	24.9 ± 1.4	25.8 ± 1.2	0.350
**Active smoking**	2 (9.1)	1 (5.9)	1.000
**Maintenance medication**			0.501
***None***	7 (31.8)	3 (17.6)	
***Thiopurines***	4 (18.2)	4 (23.5)	
***Mesalamine***	4 (18.2)	2 (11.8)	
***TNF-antagonists***	3 (13.6)	6 (35.3)	
***Combination therapy***	4 (18.2)	2 (11.8)	
**Montreal, Localization**			0.194
***L1 (ileal)***	11 (52.4)	4 (23.5)	
***L2 (colonic)***	3 (14.3)	4 (23.5)	
***L3 (ileocolonic)***	7 (33.3)	9 (52.9)	
**Montreal, Behavior**			0.322
***B1 (non stricturing*, *non penetrating)***	14 (66.7)	8 (47.1)	
***B2 (stricturing)***	5 (23.8)	8 (47.1)	
***B3 (penetrating)***	2 (9.5)	1 (5.9)	
**Ileocecal resection**	8 (36.4)	5 (29.4)	0.740
**Hemoglobin (mmol/l)**	8.4 ± 0.2	8.4 ± 0.2	0.787
**CRP (mg/l)**	2.1 ± 0.6	8.2 ± 2.4	0.002*
**ESR (mm/h)**	12.5 ± 2.2	23.6 ± 4.2	0.017*
**WBC (x10^9^/l)**	6.9 ± 0.5	6.9 ± 0.4	0.723
**Thrombocytes (x10^9^/l)**	264 ± 13	308 ± 17	0.229
**ASAT (U/l)**	22 ± 1	23 ± 3	0.977
**ALAT (U/l)**	18 ± 2	23 ± 5	0.580
**Creatinine (μmol/l)**	68 ± 2	70 ± 3	0.681

BMI, body mass index; HBI, Harvey Bradshaw index; CRP, C-reactive protein; ESR, erythrocyte sedimentation rate; WBC, white blood cell count; ASAT, aspartate aminotransferase; ALAT, alanine aminotransferase. Data are presented as numbers (proportions, n (%)) or mean ± SE. Differences between groups were tested with Mann-Whitney U-test for continuous variables and Fisher’s exact test for nominal variables.

*P value < 0.05 was considered statistically significant;

**P value < 0.01.

In Table 2, serum biomarker levels (pg/mL) are shown as median values with interquartile ranges (IQR) for both groups. All detected molecules are grouped in different experimental panels (Pro-inflammatory panel, Cytokine panel, Chemokine panel, Angiogenesis panel and Vascular injury panel).

**Table 2:** Median (IQR) of serum levels of all detected molecules (pg/mL) in Crohn’s disease patients with ‘normal’ (< 200 mg/kg) and increased (> 200 mg/kg) fecal calprotectin levels.

Detected molecules	Calprotectin < 200 mg/kg	Calprotectin > 200 mg/kg	*P*-value
**Pro-inflammatory panel**			
IFN-γ	7.80 (4.09–19.72)	23.00 (10.13–38.72)	< 0.05*
IL-1β	0.05 (0.04–0.08)	0.05 (0.03–0.13)	0.945
IL-2	0.18 (0.12–0.23)	0.17 (0.10–0.25)	0.932
IL-4	0.04 (0.02–0.07)	0.04 (0.03–0.06)	0.777
IL-6	0.78 (0.39–1.15)	1.29 (0.93–1.88)	< 0.01**
IL-8	11.91 (7.20–15.43)	12.63 (6.35–68.62)	0.955
IL-10	0.32 (0.19–0.44)	0.37 (0.24–0.74)	0.321
IL-12p70	0.09 (0.03–0.26)	0.07 (0.04–0.18)	0.624
IL-13	0.56 (0.42–0.91)	0.77 (0.55–1.79)	0.153
TNF-α	3.13 (2.50–3.67)	3.49 (2.82–4.79)	0.095
**Cytokine panel**			
GM-CSF	0.16 (0.07–0.29)	0.15 (0.07–0.24)	0.784
IL-5	0.26 (0.16–0.57)	0.26 (0.19–0.54)	0.883
IL-7	19.95 (13.57–28.08)	18.93 (10.18–27.09)	0.552
IL-12/23p40	118.18 (74.94–204.08)	158.38 (125.23–222.36)	0.157
IL-15	3.04 (2.31–5.66)	2.87 (2.44–5.74)	0.777
IL-16	284.06 (203.49–316.63)	285.97 (195.06–327.77)	0.755
IL-17A	5.25 (3.04–9.27)	8.22 (5.11–11.85)	0.058
TNF-β	0.49 (0.34–0.55)	0.55 (0.42–0.66)	0.051
**Chemokine panel**			
Eotaxin	266.32 (179.61–351.18)	240.14 (190.62–356.28)	0.955
MIP-1β	90.12 (73.41–172.00)	80.40 (47.64–129.26)	0.322
Eotaxin-3	20.29 (15.72–30.32)	15.57 (11.79–22.39)	0.101
TARC	242.32 (138.99–512.79)	267.11 (156.86–359.57)	0.799
IP-10	2.99x10^3^ (2.40x10^3^-4.62x10^3^)	3.60x10^3^ (2.25x10^3^-4.66x10^3^)	0.590
MIP-1α	16.27 (13.31–20.75)	16.75 (12.69–24.30)	0.966
MCP-1	217.13 (166.20–357.20)	261.95 (188.06–357.75)	0.630
MDC	1.33x10^3^ (1.02x10^3^-1.69x10^3^)	1.28x10^3^ (1.07x10^3^-1.66x10^3^)	0.977
**Angiogenesis panel**			
VEGF	469.52 (236.89–726.63)	382.16 (223.83–896.71)	0.755
VEGF-C	569.55 (442.96–737.47)	462.11 (375.45–628.34)	0.141
VEGF-D	1.07x10^3^ (770.27–1.47x10^3^)	901.29 (799.81–1.41x10^3^)	0.777
Tie-2	804.11 (652.49–969.74)	708.28 (626.90–845.61)	0.213
Flt-1	147.69 (126.14–167.65)	144.15 (129.16–171.30)	0.932
PIGF	6.28 (5.45–7.40)	5.80 (5.10–7.85)	0.876
bFGF	5.08 (2.05–21.97)	9.12 (2.29–27.11)	0.745
**Vascular injury panel**			
SAA	4.78x10^6^ (2.84x10^6^- 8.87x10^6^)	9.02x10^6^ (4.64x10^6^- 2.87x10^7^)	< 0.05*
CRP	1.25x10^6^ (6.03x10^5^- 4.67x10^6^)	6.79x10^6^ (3.61x10^6^- 2.64x10^7^)	< 0.01**
VCAM-1	8.28x10^5^ (7.83x10^5^-9.64x10^5^)	8.91x10^5^ (7.67x10^5^-1.02x10^6^)	0.671
ICAM-1	4.97x10^5^ (4.27x10^5^-6.00x10^5^)	5.63x10^5^ (4.99x10^5^-7.06x10^5^)	0.062

*P value < 0.05 was considered statistically significant;

**P value < 0.01.

In the Pro-inflammatory panel, concentrations of IFN-γ and IL-6 were significantly elevated in CD patients with ‘increased’ fecal calprotectin levels as compared to patients with ‘normal’ fecal calprotectin levels (IFN-γ: 23.00 pg/mL (IQR: 10.13–38.72) vs. 7.80 pg/mL (IQR: 4.09–19.72) (*P* < 0.05); IL-6: 1.29 pg/mL (0.93–1.88) vs. 0.78 pg/mL (0.39–1.15) (*P* < 0.01). In addition, in the Vascular injury panel, we found significantly higher concentrations of SAA and CRP in patients with increased fecal calprotectin levels as compared to the group with normal fecal calprotectin levels (SAA: 9.02x10^6^ pg/mL (IQR: 4.64x10^6^-2.87x10^7^) vs. 4.78x10^6^ pg/mL (2.84x10^6^-8.87x10^6^) (*P* < 0.05); CRP: 6.79x10^6^ pg/mL (IQR: 3.61x10^6^-2.64x10^7^) vs. 1.25x10^6^ pg/mL (IQR: 6.03x10^5^-4.67x10^6^) (*P* < 0.01)). In the Cytokine panel, Chemokine panel and Angiogenesis panel, no significant differences in serum biomarker concentrations were observed between both groups. Interestingly, we also observed elevated concentrations of IL-17A and TNF-β in patients with increased fecal calprotectin levels, but these were borderline non-significant (*P* = 0.058 and *P* = 0.051, respectively).

**Fig 1:**
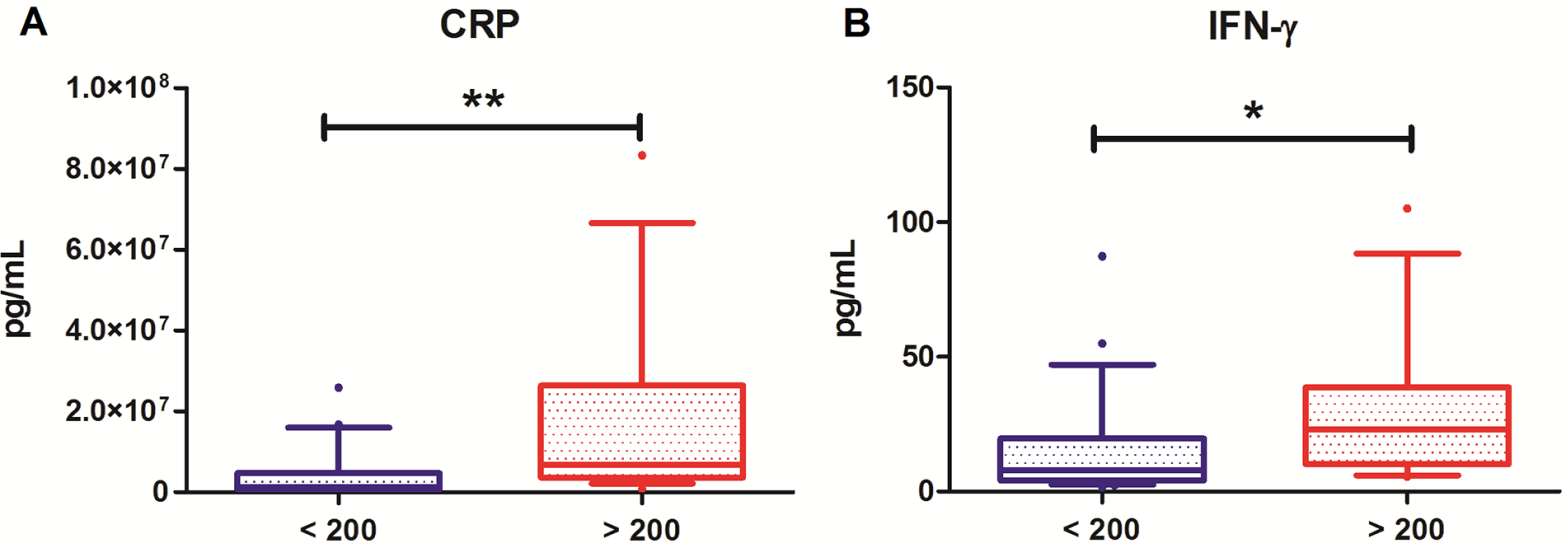
Distribution of serum biomarker levels in patients with normal (blue, < 200 mg/kg) and increased (red, > 200 mg/kg) fecal calprotectin levels, shown in boxplots. (A) Serum CRP levels (pg/mL). (B) Serum IFN-γ levels (pg/mL). *P < 0.05; **P < 0.01.

**Fig 2:**
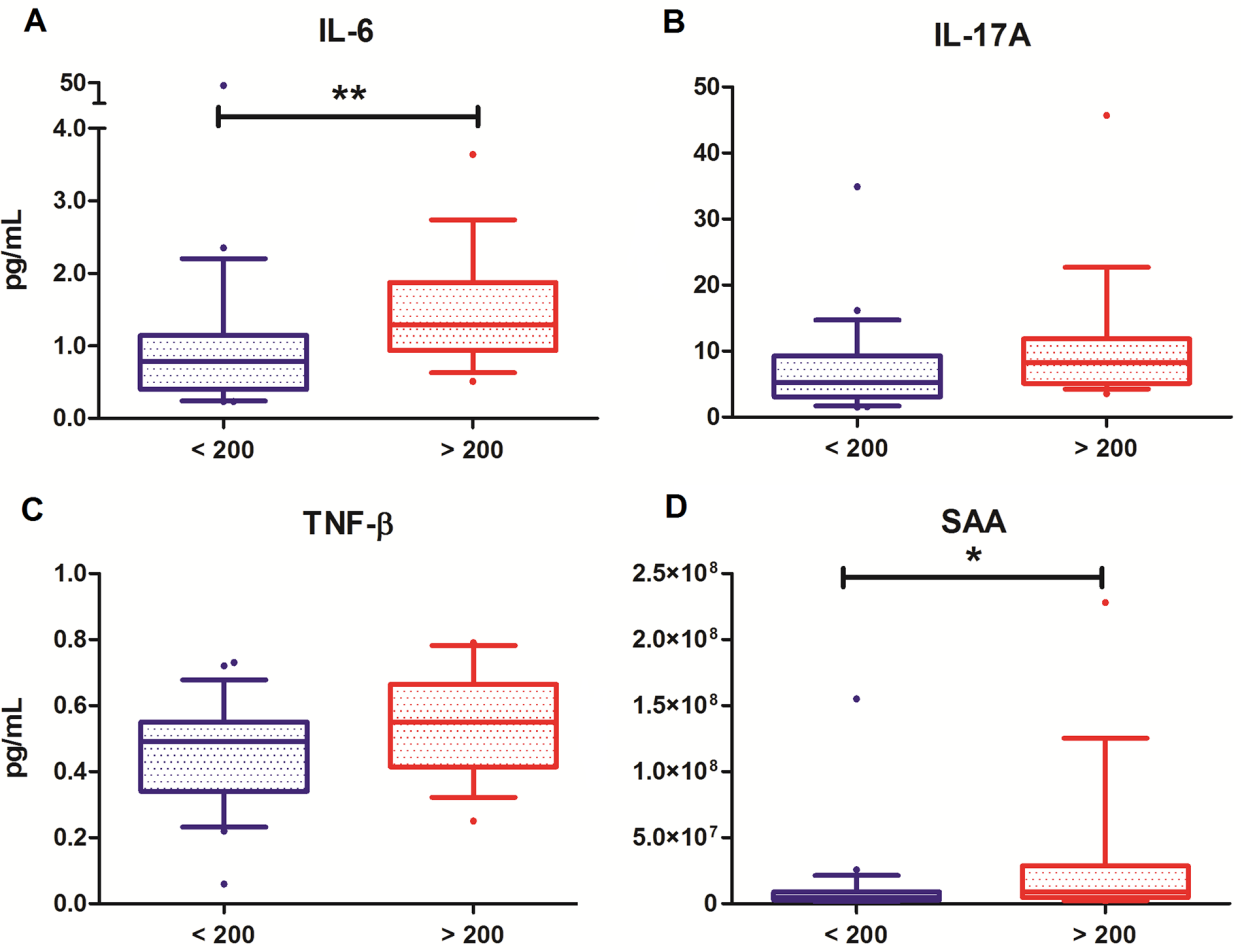
Distribution of serum biomarker levels in patients with normal (blue, < 200 mg/kg) and increased (red, > 200 mg/kg) fecal calprotectin levels, shown in boxplots. (A) Serum IL-6 levels (pg/mL). (B) Serum IL-17A levels (pg/mL). (C) Serum TNF-β levels (pg/mL). (D) Serum SAA levels (pg/mL). *P < 0.05; **P < 0.01.

Distributions of the significantly correlating serum biomarkers between groups are shown in Fig 1 (IFN-γ and CRP, *P* < 0.01) and Fig 2 (IL-6, IL-17A, TNF-β and SAA, *P* < 0.05). A highly significant positive correlation was observed between the pro-inflammatory cytokines IFN-γ (*ρ* = 0.523, *P* < 0.01, Fig 1A) and CRP (*ρ* = 0.511, *P* < 0.01, Fig 1B) and fecal calprotectin levels. Significant, but less strong correlations were demonstrated between IL-6 (*ρ* = 0.403, *P* < 0.05, Fig 2A), IL-17A (*ρ* = 0.352, *P* < 0.05, Fig 2B), TNF-β (*ρ* = 0.396, *P* < 0.05, Fig 2C) and SAA (*ρ* = 0.323, *P* < 0.05, Fig 2D), and fecal calprotectin levels. No significant correlations were observed between the fecal calprotectin level and the other 31 investigated cytokines, chemokines and markers for angiogenesis and vascular injury.

Table 3 provides a complete overview of all (significant and non-significant) correlations between serum concentrations of all analyzed biomarkers and fecal calprotectin levels.

**Table 3:** Correlations of fecal calprotectin levels (mg/kg) with serum levels of all detected molecules (pg/mL) in patients with Crohn’s disease.

Detected molecules	Spearman’s *ρ*	*P*-value
**Pro-inflammatory panel**		
IFN-γ	0.523	< 0.01**
IL-1β	0.037	0.835
IL-2	-0.063	0.702
IL-4	0.111	0.526
IL-6	0.403	< 0.05*
IL-8	0.033	0.844
IL-10	0.135	0.411
IL-12p70	0.071	0.676
IL-13	0.101	0.647
TNF-α	0.255	0.117
**Cytokine panel**		
GM-CSF	0.004	0.981
IL-5	0.148	0.374
IL-7	-0.005	0.974
IL-12/23p40	0.214	0.191
IL-15	0.088	0.596
IL-16	-0.018	0.914
IL-17A	0.352	< 0.05*
TNF-β	0.396	< 0.05*
**Chemokine panel**		
Eotaxin	-0.003	0.986
MIP-1β	-0.126	0.446
Eotaxin-3	-0.213	0.199
TARC	-0.112	0.499
IP-10	0.207	0.207
MIP-1α	0.094	0.569
MCP-1	-0.025	0.878
MDC	0.088	0.593
**Angiogenesis panel**		
VEGF	0.019	0.907
VEGF-C	-0.256	0.116
VEGF-D	0.004	0.980
Tie-2	-0.205	0.211
Flt-1	0.087	0.599
PIGF	0.023	0.888
bFGF	-0.007	0.966
**Vascular injury panel**		
SAA	0.323	< 0.05*
CRP	0.511	< 0.01**
VCAM-1	0.148	0.367
ICAM-1	0.304	0.060

*P value < 0.05 was considered statistically significant;

**P value < 0.01.

## Discussion

In this study, we show that increased fecal calprotectin levels—a broadly applied marker for the assessment of disease activity in CD patients—highly significantly correlate with elevated serum IFN-γ and CRP levels. Moreover, significant correlations were observed between serum levels of IL-6, IL-17A, TNF-β and SAA and fecal calprotectin levels. These findings indicate that identification of biomarker profiles might serve as an additional approach to determine inflammatory disease activity in CD patients.

Our data not only provide correlations between fecal calprotectin levels and inflammatory markers in the systemic circulation, but also confirm that Th1 responses are important in CD patients with active disease since IFN-γ levels were significantly elevated, as well as serum TNF-α levels, although the latter did not reach statistical significance. Furthermore, an interesting additional finding is the borderline non-significant enhancement of serum IL-17A levels (*P* = 0.058) in CD patients with increased fecal calprotectin levels. IL-17A has been shown to be a driving effector response for colitis in mice. However, transfer of T-cells from IL-17-deficient mice induced severe colitis in mice recipients and enhanced IFN-γ-producing T-cells. [20] Likewise, in humans, it is less clear whether IL-17 has a protective role or induces an effector response. [21,22] In our study, we found elevated IL-17A levels specifically in CD patients with increased fecal calprotectin levels, suggesting that it is a marker for inflammatory disease activity instead of a protective marker. Interestingly, we also found that the Th17-stimulating cytokines IL-6 and TNF-β were enhanced in the group with increased fecal calprotectin levels, further supporting that an activated Th17 response is involved in these patients in addition to the Th1 response.

Our findings on the association between C-reactive protein (CRP) and fecal calprotectin levels corroborate the findings of other studies. [23,24] CRP is an acute-phase protein, mainly produced by hepatocytes in response to systemic inflammation. Production occurs after stimulation by IL-6, IL-1β and TNF-α. In clinical practice, CRP is used as a general biomarker for inflammation and is therefore commonly applied to monitor the disease course of CD. CRP rapidly increases after an acute-phase stimulus and has a short half-life, making CRP an useful marker for acute inflammatory events in CD. [25] In CD, active disease is significantly associated with both elevated CRP and fecal calprotectin levels. [14,26] Elevated CRP levels at diagnosis are predictive for the requirement of future surgical interventions in CD and therefore for disease severity. [27] Also, increased CRP levels prior to treatment with the TNF-α-antagonist infliximab were predictive of a higher response rate and treatment success. [25,28] Additionally, our data demonstrate that enhanced serum CRP levels correlate with inflammatory disease activity, as it was specifically enhanced in the CD patient group with increased fecal calprotectin levels.

In the present study, serum IFN-γ levels were significantly elevated in CD patients with increased fecal calprotectin levels, as compared to patients with remissive disease. Likewise, it has previously been observed that circulating IFN-γ levels are strongly increased in IBD patients as opposed to healthy subjects. [29] IFN-γ is known for its central role in the Th1-driven immune response, which constitutes the major signaling pathway in the pathogenesis of CD. [15] IFN-γ is predominantly produced by Th1-differentiated T-cells residing in the intestinal lamina propria upon stimulation by IL-12-producing macrophages in the nearby environment. [30] Therefore, since we observe a strong correlation between fecal calprotectin levels and serum IFN-γ, this cytokine may be considered as a representative marker of endoscopic disease activity driven by Th1-cell cytokine production (IL-6, IL-12, TNF-α and IFN-γ). [15,31] Given these findings, follow-up studies are warranted to determine whether therapeutic interference in patients with increased fecal calprotectin levels accompanied by high IFN-γ and CRP levels results in improved patient outcomes.

Finally, we observed a positive correlation between fecal calprotectin levels and serum amyloid A (SAA), an acute-phase protein implicated in multiple chronic inflammatory diseases and commonly found to be elevated in CD patients. [32] SAA is an apolipoprotein of high-density lipoproteins (HDL) and plays a role in cholesterol transport to and from sites of inflammation. [33] It has previously been demonstrated that SAA correlates well with other acute-phase reactants, such as CRP and alpha-1-antichymotrypsin (alpha-1-ACT) and is suggested to be helpful in monitoring CD disease activity. [24,34]

In the present study, the concentrations of a large number of biomarkers have been assessed using a highly-sensitive electrochemiluminescence (ECL) assay and were directly correlated to the fecal calprotectin level. To our best knowledge, no studies have focused on the direct correlations between fecal calprotectin levels and serum biomarker levels with such a wide dynamic range of detection, providing us with high sensitivity measurements of cytokines, chemokines and markers for angiogenesis and vascular injury. However, a limitation of this study is that no endoscopic results were available for this patient cohort, which is generally considered as gold standard for demonstrating disease activity in CD. [35,36] In clinical practice, it is challenging to identify an acute disease exacerbation, since its clinical presentation is often highly non-specific. Regularly, CD patients have concomitant symptoms that might be attributed to functional bowel syndromes, stricturing CD or gastro-intestinal infections. In contrast, other patients that do not present with any disease symptoms, may still show an active intestinal inflammation as determined by endoscopic investigation.

In this study, we have identified multiple candidate serum biomarkers to quantify the severity of a flare in patients with confirmed CD. This could also lead to means to distinguish inflammatory from non-inflammatory disease flares. Future prospective studies are warranted in which CD patients undergoing an endoscopic disease evaluation will be included in order to confirm that indeed only in patients with elevated Th1- and Th17-associated cytokines, inflammatory flares have occurred. Altogether, this may lead to an advanced prediction model for detecting disease exacerbations, based on a combined array of currently identified Th1- and Th17-associated cytokines. Subsequently, the predictive accuracy of this model could be compared with the distinctive power of endoscopic disease activity measurement.

In conclusion, we show that a positive correlation exists between multiple Th1- and Th17-associated cytokines and fecal calprotectin levels, presenting additional non-invasive candidate biomarkers for clinical use in CD patients. Moreover, these candidate biomarkers could be of value in monitoring and treating disease activity in CD. Future studies should aim to further assess the diagnostic potential of a distinct biomarker profile in relation to endoscopic activity measures.
